# Contralateral tension pneumothorax during video-assisted thoracic surgery for lung cancer in a patient with obesity and rib fractures: a case report and review of the literature

**DOI:** 10.1186/s13256-020-02556-w

**Published:** 2020-11-14

**Authors:** Sakiko Kumata, Katsunari Matsuoka, Shinjiro Nagai, Mitsuhiro Ueda, Yoshinori Okada, Yoshihiro Miyamoto

**Affiliations:** 1 Department of Thoracic Surgery, Institute of Development, Aging and Cancer, Tohoku University, 4-1 Seiryomachi, Aobaku, Sendai, 980-8575 Japan; 2 Department of Thoracic Surgery, National Hospital Organization Himeji Medical Center, Himeji, Japan

**Keywords:** Tension pneumothorax, Contralateral pneumothorax, One-lung ventilation, Lung cancer, Video-assisted thoracic surgery

## Abstract

**Background:**

Intraoperative contralateral pneumothorax during one-lung ventilation is a rare but life-threatening complication. Although the exact incidence is unknown, only 14 cases with this complication have been reported until now.

**Case presentation:**

A 67-year-old Japanese man with a weight of 80 kg, height of 162.2 cm, and body mass index of 30.4 kg/m^2^ underwent three-port video-assisted thoracic surgery for lung cancer with one-lung ventilation. He had suffered from traumatic right rib fractures 6 weeks before the referral. Fifteen minutes before the end of the surgery, the systolic blood pressure suddenly dropped to about 50 mmHg, which was immediately recovered by intravenous injection of phenylephrine. This episode occurred during chest closure after the completion of the left upper lobectomy, and one-lung ventilation was soon switched to two-lung ventilation. Contralateral tension pneumothorax was noted by the postoperative chest x-ray. As the patient was complicated with obesity and a past history of rib fractures, increased airway pressure during one-lung ventilation related to obesity together with the persistent compression of the visceral pleura by the fractured ends of the ribs was considered to be the factors responsible for this critical complication.

**Conclusions:**

Patient backgrounds such as obesity and past history of rib fractures should be noted carefully as risk factors for intraoperative contralateral pneumothorax during one-lung ventilation. We present the clinical course and discuss the mechanism of development of this potentially life-threatening complication in the present case with a review of the literature.

## Background

Pneumothorax during general anesthesia is a rare event that occurs in only 0.0012% of patients in Japan [1]. Intraoperative contralateral pneumothorax during one-lung ventilation (OLV) appears to be even more rare with only 14 cases reported until now. Furthermore, among these 14 cases, only 2 case reports were found with minimally invasive surgeries including a case with robotic-assisted thoracic surgery (RATS) and the other with video-assisted thoracic surgery (VATS). As OLV is an indispensable technique for lung resection with RATS and VATS, and pneumothorax during OLV is a life-threatening complication that requires prompt diagnosis and treatment, anesthesiologists and surgeons should keep the risk of this complication in mind during surgery despite its rarity. It is also important to be aware of preoperative risk factors for this critical complication.

## Case presentation

**Fig. 1:**
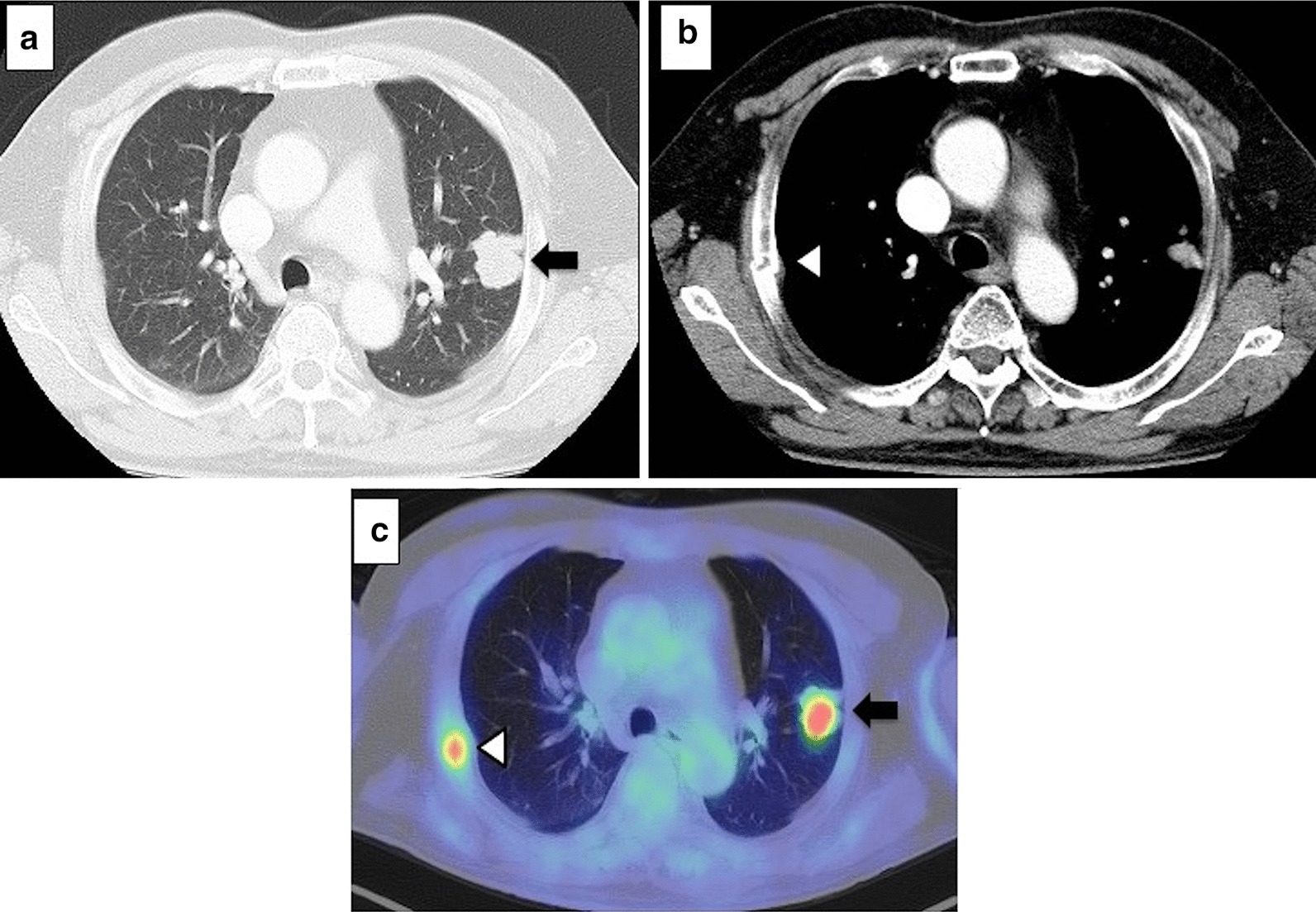
**a**, **b** Chest computed tomography shows a mass located in left upper lobe (←) and no pulmonary bullae. Fractures of the right third, fourth, and fifth ribs were noted (white left-pointing triangle). **c** Fluorine-18-fluorodeoxyglucose positron emission tomography/computed tomography demonstrated uptakes in the tumor of the left upper lobe (←) and the site of rib fractures

A 67-year-old Japanese man with weight of 80 kg, height of 162.2 cm, and body mass index (BMI) of 30.4 kg/m^2^ was referred to our hospital for examination of a pulmonary mass in the left upper lobe. He had suffered from traumatic right rib fractures 6 weeks before the referral. Chest computed tomography (CT) on admission demonstrated a mass 32 mm in diameter in the anterior segment of the left lung (S3) (Fig. 1a) without obvious swelling of hilar or mediastinal lymph nodes. The CT also showed right 3rd–5th rib fractures, which were almost healed, but the fractured ends projected into the chest cavity (Fig. 1b). Bilateral lung fields showed no emphysema or bullae. Fluorine-18-fluorodeoxyglucose positron emission tomography/CT demonstrated uptakes in the tumor of the left upper lobe and the site of the rib fractures (Fig. 1c), and no uptake was found in hilar or mediastinal lymph nodes. The clinical stage of the patient was judged to be cT2aN0M0 (stage 1B). He had no specific comorbidities except for obesity and no smoking history. The mass in his left lung was diagnosed as adenocarcinoma by transbronchial biopsy. Preoperative pulmonary function test showed a mild obstructive ventilatory impairment with a forced vital capacity (FVC) of 2.67 L (80% of predicted), a forced expiratory volume in one second (FEV_1_) of 1.84 L (68.0% of predicted), and 68.9% of FEV_1_/FVC.

**Fig. 2:**
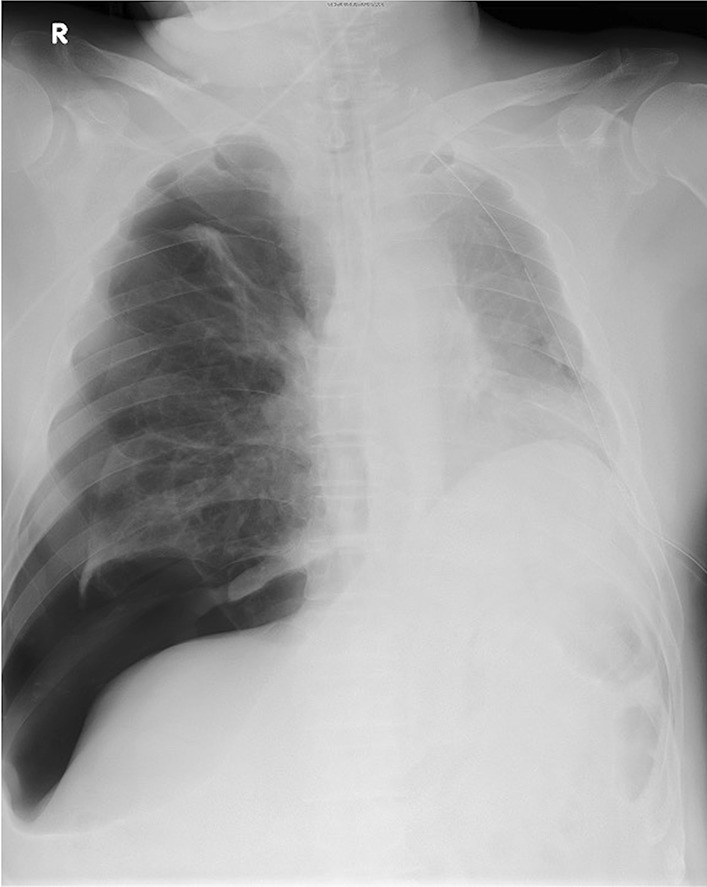
Chest x-ray using a portable s-ray system reveals a right-sided tension pneumothorax after the surgery

After the induction of general anesthesia, a right-sided 37-French double-lumen endotracheal tube (DLT, BronchoCath®, Mallinckrodt Medical, Ireland) was inserted. The position of the DLT was adjusted using bronchofiberscopy. The patient received volume-controlled ventilation appropriated for the physique. The tidal volume during OLV was set to 200 mL, and the peak inspiratory pressure with this tidal volume was about 35 cmH_2_O. The patient was placed in the right decubitus position and underwent left upper lobectomy under three-port VATS. Approximately 2 hours after the start of the surgery and during the closure of the wound, the patient’s blood pressure suddenly decreased to 50 mmHg. The arterial oxygen saturation of pulse oximetry (SpO_2_) and heart rate remained stable. The blood pressure improved immediately after the intravenous injection of phenylephrine. After placing a chest drain tube in the surgical side, a few minutes after the transient hypotension, OLV was switched to two-lung ventilation as usual. The operation was completed with the operation time of 137 minutes. The patient was rotated to the supine position, and portable chest x-ray photography was performed in the operation room, which revealed a right-sided tension pneumothorax (Fig. 2). The DLT was located in the proper position. Immediately, a chest drain tube was inserted into the right chest cavity. A little air leak was observed through the right-sided drain tube, which fortunately stopped spontaneously by the next day. The right-sided drain tube was removed on day 1 and the left-sided tube on day 2 after surgery. He left the hospital on the 8th postoperative day.

## Discussion and conclusions

Pneumothorax during general anesthesia is generally caused by the rupture of bullae and iatrogenic trauma of thoracic soft tissue by surgical procedures, intubation, or catheter insertion [1]. In addition, contralateral pneumothorax during OLV can occur in association with inappropriate size and incorrect position of the DLT [2]. Oversized tubes cause blunt injury of the trachea or bronchus followed by a pneumothorax. On the other hand, small tubes can be inserted too deeply, and excessive airway pressure applied to one lobe can lead to pneumothorax because of barotrauma. According to our examination of the 14 previously reported cases, the cause of pneumothorax during OLV was presumed to be barotrauma associated with a high airway pressure in nine cases, among which a size mismatch of the DLT was involved in four cases [3]. In our case, the size of the DLT was adequate, and its position was confirmed by bronchofiberscopy. During OLV in the lateral decubitus position, the dependent lung is usually under-ventilated as it is compressed by the abdominal contents and the weight of the mediastinum. The present case was complicated with obesity, which usually reduces compliance of the lung because of the increased intrathoracic and intraabdominal pressure. Thus, the peak airway pressure during OLV in obese patients generally becomes unavoidably higher than that in non-obesity cases to maintain adequate blood gas. In general, a peak airway pressure > 50 cmH_2_O and a plateau pressure > 35 cmH_2_O are reported to indicate risk of barotrauma during ventilation especially in the pathological lung [4, 5]. The peak airway pressure in the present case was about 35 cmH_2_O during OLV, which is actually high, but does not appear high enough to cause pneumothorax independently. However, our patient had a past history of rib fractures on the dependent side, and this may have caused focal pleural damage in combination with the increased airway pressure. Indeed, we previously reported a case of pneumothorax caused by costal exostosis projecting into the chest cavity [6].

Reviewing the previous 14 reports, rib fracture was not demonstrated as a cause of the complication. Similarly, in none of the four cases in which the patients’ height and body weight were described did the BMI exceed 30 kg/m^2^. However, our experience in the present case suggests that obesity and rib fracture should be recognized as risk factors for pneumothorax during OLV, given the high prevalence of rib fractures by blunt chest trauma in the elderly [7].

Since contralateral pneumothorax during OLV is a critical situation, it is important to anticipate this complication as soon as the vital signs change and before the patient falls into severe cardiopulmonary failure. The vital sign changes related to this episode included a decrease in blood pressure and/or SpO_2_, a change in heart rate, an increase in airway pressure, and a decrease in the end-tidal carbon dioxide concentration [1, 8]. Moreover, Malik *et al.* demonstrated that a macroscopic finding of intrathoracic mediastinal herniation into the thoracic cavity of the surgical side is a notable sign for intraoperative contralateral pneumothorax during OLV [9]. However, it may be more difficult to notice this sign under limited views of RATS or VATS compared with those in large thoracotomy. Ultrasonography is reported to be useful for diagnosing pneumothorax [10], but it may sometimes also be difficult to adapt to the thorax in the bottom side in the lateral decubitus position.

In our case, transient hypotension during chest closure was the only symptom that suggested contralateral pneumothorax, and two-lung ventilation was fortunately restored soon after this episode. Tension pneumothorax during OLV, however, represents a definitely life-threatening emergency, as shown by previous reports in which 1 of 14 cases died during operation [11] and another went into cardiopulmonary arrest and died several weeks later [2]. When vital sign changes suggestive of this complication are encountered, immediate restoration of two-lung ventilation and surveillance of pneumothorax by chest x-ray or ultrasonography should be considered.

## Conclusions

In summary, we present a case with contralateral tension pneumothorax during VATS with OLV in a patient with obesity and a past history of rib fractures. We propose that the backgrounds of such patients should be noted carefully as risk factors for this potentially life-threatening complication.

## Data Availability

Case report data and patient’s consent form are available.

## Abbreviations

OLV: One lung ventilation
RATS: Robotic-assisted thoracic surgery
VATS: Video-assisted thoracic surgery
CT: Computed tomography
FVC: Forced expiratory volume
FEV_1_: Forced expiratory volume in 1 second
DLT: Double-lumen endotracheal tube
SpO_2_: Oxygen saturation of pulse oximetry
